# Intrinsic Rhythmicity Predicts Synchronization-Continuation Entrainment Performance

**DOI:** 10.1038/s41598-018-29267-z

**Published:** 2018-08-06

**Authors:** Trevor McPherson, Dorita Berger, Sankaraleengam Alagapan, Flavio Fröhlich

**Affiliations:** 1 Department of Psychiatry, University of North Carolina at Chapel Hill, Chapel Hill, NC 27599 USA; 2 Department of Neurology, University of North Carolina at Chapel Hill, Chapel Hill, NC 27599 USA; 3 Department of Biomedical Engineering, University of North Carolina at Chapel Hill, Chapel Hill, NC 27599 USA; 4 Department of Cell Biology and Physiology, University of North Carolina at Chapel Hill, Chapel Hill, NC 27599 USA; 5 Neuroscience Center, University of North Carolina at Chapel Hill, Chapel Hill, NC 27599 USA; 6 Carolina Center for Neurostimulation, University of North Carolina at Chapel Hill, Chapel Hill, NC 27599 USA

## Abstract

Rhythmic entrainment—defined as a stable temporal relationship between external periodic signals and endogenous rhythmic processes—allows individuals to coordinate with environmental rhythms. However, the impact of inter-individual differences on entrainment processes as a function of the tempo of external periodic signals remain poorly understood. To better understand the effects of endogenous differences and varying tempos on rhythmic entrainment, 20 young healthy adults participated in a spontaneous motor tempo (SMT) task and synchronization-continuation tasks at three experimental tempos (50, 70, and 128 bpm; 1200, 857, and 469 ms inter onset interval (IOI)). We hypothesized that SMT task performance and tempo would influence externally paced synchronization-continuation task behavior. Indeed, intrinsic rhythmicity assessed through the SMT task predicted performance in the externally paced task, allowing us to characterize differences in entrainment behavior between participants with low and high endogenous rhythmicity. High rhythmicity individuals, defined by better SMT performance, deviated from externally paced pulses sooner than individuals with low rhythmicity, who were able to maintain externally paced pulses for longer. The magnitude of these behavioral differences depended on the experimental tempo of the synchronization-continuation task. Our results indicate that differences in intrinsic rhythmicity vary between individuals and relate to tempo-dependent entrainment performance.

## Introduction

Music therapy interventions are a cost-effective, accessible, and holistic treatment option with social, rhythmic, creative, sensorimotor, movement, and respiratory components that have the potential to improve quality of life for a diverse array of disorders^1–8^. Despite this, the literature surrounding music therapy is controversial due to the lack of standardization in clinical and research practice. Interventions range from passive listening of participant-selected music to clinician-led improvisational sessions. This has limited the pursuit of a mechanistic understanding of how music therapy functions, and what components actually produce therapeutic effects.

Rhythmicity has proven to be a particularly salient aspect of music therapy, with clinicians using rhythmic interventions to assist in motor rehabilitation, and to improve mobility for patients with Parkinson’s Disease and cerebral palsy^1–5^. These interventions are based on the idea that entrainment—defined as a stable temporal relationship between an external stimulus and an endogenous rhythm—can target physiological and sensorimotor systems to produce therapeutic benefits^2,8–11^. Motor entrainment is ubiquitous throughout human behavior, is both spontaneously initiated between individuals or the environment^12–15^, and is actively cultivated in a variety of musical and cultural contexts^16–19^. Auditory stimuli readily modulate and entrain motor movements^20–22^, supporting musical entrainment as a mechanism of treatment for motor disabilities. Auditory stimuli have also been shown to produce high precision motor responses^23,24^ and faster reactions times in comparison to other stimulus modalities^25,26^, demonstrating that the auditory system, due to its high temporal fidelity, is particularly well adapted for facilitating motor entrainment.

Behavioral entrainment of human motion is typically assessed through sensorimotor synchronization (SMS) tasks, in which participants synchronize their movements with a periodic stimulus. Most of the SMS literature is based on a finger tapping paradigm^21,22^, and the variability in the inter-onset-interval (IOI) of taps and asynchrony between auditory stimuli and taps are typically used to assess behavioral performance in SMS tasks. In these tasks, the pulse is the isochronous rhythm that participants entrain with and produce, and the tempo is the frequency of the stimulus and produced rhythm. A derivative of the SMS paradigm is the synchronization-continuation task, in which participants initially entrain with an external periodic stimulus that is later removed, leaving the participants to maintain the pulse without the rhythmic prompt. Thus, in synchronization-continuation tasks there is no external signal that sustains the pulse and participants must rely on internal mechanisms to maintain entrainment to the given tempo. Another sensorimotor task—the spontaneous motor tempo (SMT) task—examines how individuals produce spontaneous pulses. Here, participants are instructed to continuously generate a pulse at a constant tempo of their choice, allowing for the examination of participant-driven preferences in tempo. This is not an entrainment task as no external pulse is provided, making the SMT task reflective of the endogenous processes that are presumed to underlie the ability to rhythmically entrain. Previous studies have described a relationship between SMT and optimal entrainment performance in other tasks^27,28^, suggesting that endogenous characteristics, inherent to individuals, can alter performance in SMS tasks and bias individuals to preferentially coordinate with optimal tempos. An assay to calculate an optimal SMS tempo for individuals could influence music therapy practice, as rhythmic interventions could then be tailored to accommodate the differing tempos that patients are best suited to entrain with.

When examining SMT, Styns *et al*.^27^ examined the synchronization of spontaneous walking tempos to externally paced musical tempos, while Delevoye-Turrell *et al*.^28^ found that IOI and asynchrony were lowest when performing synchronization-continuation tasks closest to SMT. SMT pulse performance accuracy, defined through SMT tasks, and its relationship to performance in synchronization-continuation paradigms has not been examined. We propose that individual differences in intrinsic rhythmicity can be seen in the temporal consistency of SMT pulses, and that differences in intrinsic rhythmicity will be reflected in synchronization-continuation task entrainment. The impact of intrinsic rhythmicity on differential entrainment tempos has also not been studied. By conducting synchronization-continuation tasks at a variety of tempos we intended to examine how different frequencies interact with intrinsic rhythmicity to produce behavioral entrainment characteristics. Ultimately, we hope to clarify the relationship between endogenous differences and variable frequencies on the behavioral processes that underlie rhythmic entrainment, allowing for a more comprehensive understanding of rhythmic entrainment.

The study we present in this paper investigated the effect of intrinsic rhythmicity and variable frequencies upon entrainment behavior through SMT and synchronization-continuation paradigms. The intrinsic rhythmicity of each participant was assessed through an SMT drumming task, which was followed by synchronization-continuation tasks at three experimental tempos—50, 70, and 128 beats per minute (bpm); 1200, 857, and 469 ms inter onset interval (IOI). Through individual differences in intrinsic rhythmicity, we characterized differences in synchronization-continuation task performance, and differences across tempos. We hypothesized that the intrinsic rhythmicity of a person influences synchronization-continuation task behavior, and that this process occurs differentially at various tempos. In contrast to the extensive tapping literature, we implemented a naturalistic drumming task representative of an active music therapy intervention model. Previous work has implemented naturalistic drumming in rhythm research^10,29,30^ and contrasted it against finger taping^31,32^, revealing that finger tapping produces greater variability in motor synchronization than naturalistic drumming. Drumming engages the skeleto-muscular system in more comprehensive ways than finger tapping tasks and employs a range of motion that individuals regularly employ on a daily basis, making it more representative of holistic entrainment abilities^33–35^. This task engages sensorimotor entrainment that would occur in a music therapy context, making it crucial for assessing ecologically valid measurements of entrainment and potential translational therapeutic applications of our research.

## Results

### Distribution of entrainment and tempo data

SMT ranged from 62–122 bpm for most participants, with one individual producing an SMT of 189 bpm. Phase locking value (PLV) was used to assess the overall temporal organization of the drum hits produced by each participant. Here, PLV is a circular measure in which a vector in the polar plane represents the distance of each drum hit relative to the nearest previous prompt beat. Vectors for all the drum hits in each task—the SMT task and the 50 bpm (1200 ms IOI), 70 bpm (857 ms IOI), and 128 bpm (469 ms IOI) conditions of the synchronization-continuation task—were averaged together respectively to produce a resultant vector length between 0 and 1, where 1 indicates high temporal stability of drumming and 0 indicates temporal inconsistency in drumming. SMT PLV ranged from 0.0637–0.5261, and PLV in the 50 bpm, 70 bpm, and 128 bpm conditions ranged from 0.0636–0.4236, 0.0692–0.4596, and 0.0648–0.3527, respectively. Distribution plots of individual participant SMT, as well as PLV calculated from the SMT task and each of the three paced conditions—50, 70, and 128 bpm—from the synchronization-continuation task can be found in Supplementary Fig. 1.

### Behavioral entrainment did not differ between tempo conditions

The PLV for each tempo condition were compared across participants in a repeated measures ANOVA with factor tempo (within-participant, 50 bpm, 70 bpm, 128 bpm) producing no main effect (F(2,38) = 1.49, p = 0.24). Thus, behavioral entrainment as assayed through PLV did not differ between tempo conditions, implying that synchronization-continuation entrainment was similar at variable tempo frequencies.

### Intrinsic rhythmicity predicts synchronization-continuation entrainment performance

**Figure 1:**
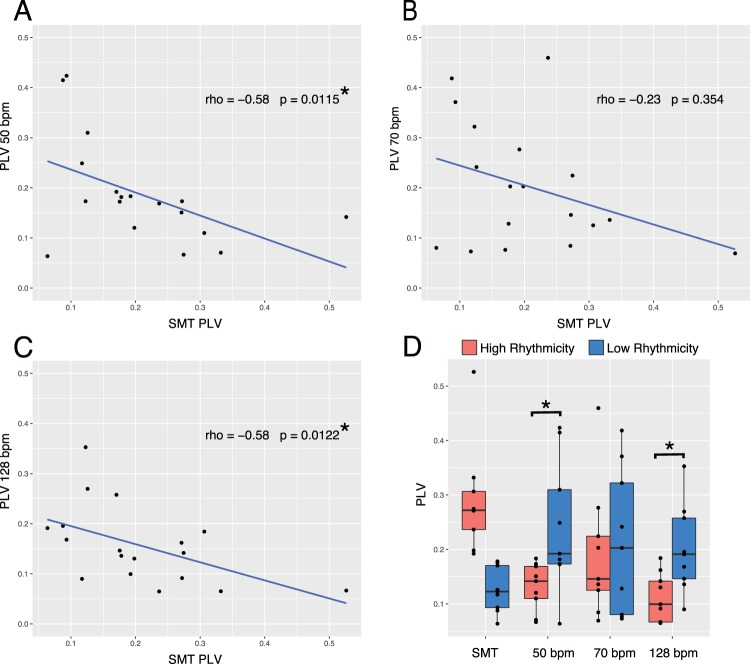
Phase Locking Value (PLV) analysis. (**A**–**C**) Scatterplots and spearman correlations for PLV from the SMT task against PLV in each of the three paced conditions (50, 70, and 128 bpm; 1200, 857, and 469 ms IOI) from the synchronization-continuation task. (**D**) After splitting participants into a high rhythmicity and a low rhythmicity group based off of SMT PLV, significant differences were found between high and low rhythmicity groups in the 50 bpm (1200 ms IOI) and 128 bpm (469 ms IOI) conditions.

Next, we compared PLV between the paced conditions—representing behavioral entrainment accuracy—and the SMT task—representing participant intrinsic rhythmicity. This allowed us to examine the relationship between the intrinsic rhythmicity of individuals and synchronization-continuation task entrainment performance. The PLV values of participants in the SMT task were correlated with the PLV values in all the other tempo conditions combined, producing a significant relationship (rho = −0.43, p = 0.0013). To examine this relationship in the context of each tempo condition, the PLV values of participants in the SMT task were correlated with their PLV values in each of the three paced conditions separately (Fig. 1A–C). Two out of the three conditions produced significant spearman correlations (50 bpm, rho = −0.58, p = 0.0115; 128 bpm, rho = −0.58, p = 0.0122). These correlations lead us to perform a median split on the SMT PLV data, grouping the participants into high rhythmicity and low rhythmicity groups, who had higher or lower SMT PLV, respectively. To verify that the significant relationship seen in the correlations would be captured by a median split, we ran a mixed model ANOVA with factors group (between-participant, high rhythmicity n = 9, low rhythmicity n = 9), and tempo (within-participant, 50 bpm, 70 bpm, 128 bpm), yielding a main effect of group (F(1,2) = 5.19, p = 0.037). This suggests that individuals with high intrinsic rhythmicity—participants who had higher PLV in the SMT task—had lower PLV in all three of the synchronization-continuation task conditions, while individuals with low intrinsic rhythmicity—who had lower PLV in the SMT task—had higher PLV in all three of the synchronization-continuation task conditions (Fig. 1D). This antagonistic performance relationship between maintaining a consistent SMT and entraining to externally paced pulses suggests that individuals have an affinity for either one or the other, and that SMT PLV could give us insight into the behavior of distinct groupings in our data. Two sample t-tests confirmed significant differences between high and low rhythmicity individuals in the 50 and 128 bpm condition, but not the 70 bpm condition (50 bpm, t(10.025) = −2.60, p = 0.026; 70 bpm, t(15.83) = −0.35, p = 0.73; 128 bpm, t(12.52) = −2.90, p = 0.013), suggesting that this effect can only be seen at certain tempos. After a Bonferroni correction (0.05/3, adjusted alpha = 0.0167) the high and low rhythmicity t-tests comparison in the 128 bpm condition remained significant.

### SMT does not predict performance in synchronization-continuation tasks

Previous studies have shown that SMT can predict behavioral entrainment performance in synchronization-continuation tasks, where synchronization-continuation entrainment accuracy is greatest at tempos that are closes to individual participant SMT^27,28,36^. To assess this with our data, we took the SMT for each participant and paired it with the tempo condition that was closest to it. If the PLV for a participant in this tempo condition was greater than that of the other two conditions, then this would indicate that the tempo closest to SMT was most accurately entrained with, and that SMT could be used to predict behavioral entrainment performance. We found this to be true in only 7 of our 18 participants, and a Chi-squared test for independence (X^2^(1, N = 18) = 0.89, p = 0.35) revealed an inconsistent relationship between SMT and preferential behavioral entrainment performance.

### Faster tempos increase continuation entrainment variability

**Figure 2:**
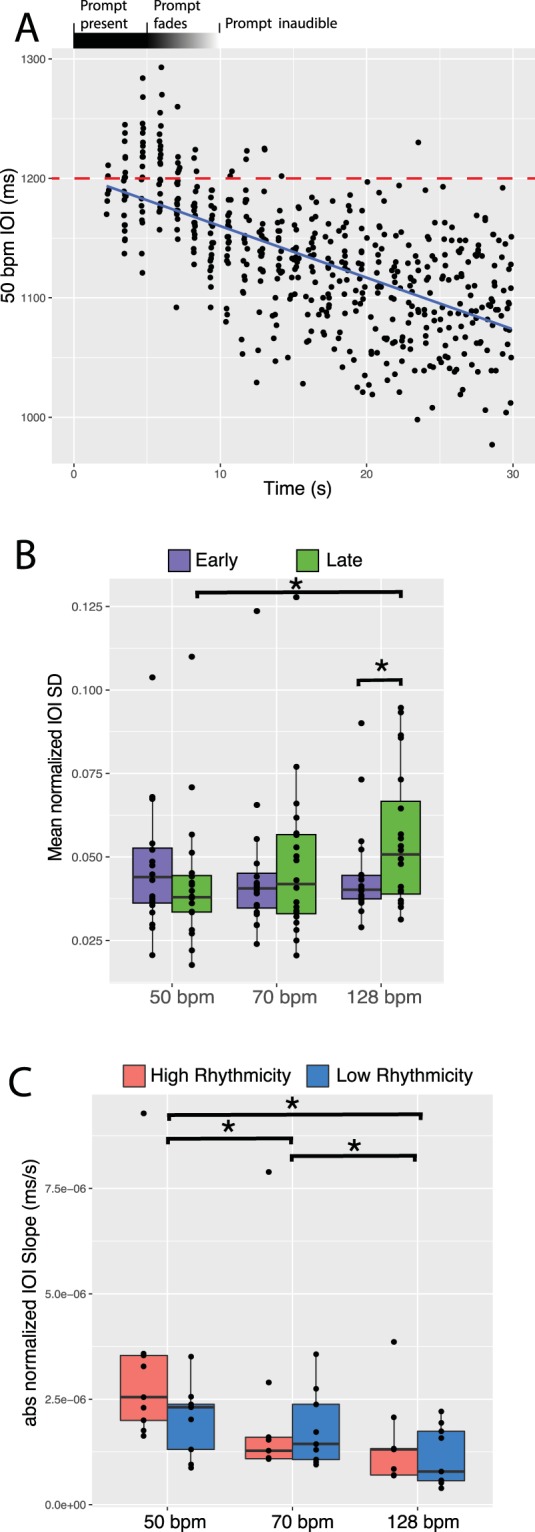
Inter Onset Interval (IOI) analysis. (**A**) One example of the IOI time course plots generated at each experimental tempo (50, 70, and 128 bpm; 1200, 857, and 469 ms IOI) for each participant. Here, the 50 bpm (1200 ms IOI) IOI time course data for one participant is plotted and fitted with a linear regression. Slopes were extracted from linear regressions of the data of each participant in each tempo conditions. The red dashed line indicates the IOI of the externally paced prompt—1200 ms. (**B**) Normalized IOI values were split into early and late groups, depending on whether they fell in the first or last 15-seconds of each trial. This allowed for a comparison of the synchronization vs. continuation entrainment phases of the synchronization-continuation task. A significant interaction between tempo and group demonstrates that faster tempos cause more variability during continuation entrainment. (**C**) The absolute values of extracted normalized IOI slopes were used to compare how high and low rhythmicity groups deviated from the paced pulse during the transition to continuation entrainment. A main effect for tempo demonstrated that slower tempos lead participants to deviate more from the original prompt frequency.

Inter onset interval (IOI) values measured the duration between each drum hit and the previous drum hit played by the participant. IOI values were used to investigate the temporal consistency of drum hits over the duration of trials. We generated IOI time course plots for each participant and tempo condition to visualize how the IOI values of participants changed over the timespan of trials (one example IOI time course plot shown in Fig. 2A). IOI values were normalized with respect to tempo to account for the differing length of IOI values due to differing tempo condition, allowing for a comparison across conditions. Trial data was split into early and late sections, comprising the normalized IOI values within the first and last 15-seconds of the trials, representing the individual synchronization and continuation sections of the synchronization-continuation task respectively. A mixed model ANOVA with factors group (within-participant, early, late) and tempo (within-participant, 50 bpm, 70 bpm, 128 bpm) revealed a group-tempo interaction (F(2,38) = 4.03, p < 0.026). To understand how tempo contributed to this interaction, we ran two one-way repeated measures ANOVAs with the factor tempo (within-participant, 50 bpm, 70 bpm, 128 bpm) for both early and late group data. A main effect was found for tempo in the late group ANOVA (F(2,38) = 38.87, p < 0.030) while no effects were found in the early group. Pairwise t-tests comparing the different tempo conditions in the late group revealed significant differences between the 50 bpm and 128 bpm tempos (t(19) = −2.69, p < 0.014). After a Bonferroni correction (0.05/3, adjusted alpha = 0.0167) the 50 and 128 bpm t-tests comparison remained significant. To understand how early vs. late groupings contributed to the previous interaction, three paired t-tests were run comparing early to late groups at each tempo condition, producing a significant difference in the 128 bpm condition (t(19) = −2.48, p = 0.023). After a Bonferroni correction (0.05/3, adjusted alpha = 0.0167) this result was not significant. Together, these results indicate that IOI variability is similar across tempo conditions initially during the synchronization portion—the first 15 seconds—of the synchronization-continuation task, yet at faster tempo conditions IOI variability increases in the continuation portion—the latter 15 seconds—of the task (Fig. 2B).

### Externally paced pulses are more difficult to maintain at slower tempos

Next, the time-course of the IOI deviations was examined in terms of high and low rhythmicity groups, and tempo condition. To do this, we fit each normalized IOI time-course plot with a linear regression and extracted the slopes of each regression line (one example IOI time course plot shown in Fig. 2A). The absolute values of the slopes were taken, making the values representative of deviation from the prompt pulse. IOI slopes were split into high and low rhythmicity groups in each tempo condition, and a mixed model ANOVA was run, with factors group (between-participant, high rhythmicity (n = 9), low rhythmicity (n = 9)), and tempo (within-participant, 50 bpm, 70 bpm, 128 bpm) revealing a main effect of tempo (F(1,2) = 15.75, p < 0.0001). Paired sample t-tests revealed significant differences between all tempo conditions (50–70 bpm, t(17) = 3.05, p = 0.0072; 50–128 bpm, t(17) = 4.74, p < 0.001; 70–128 bpm, t(17) = −2.77, p = 0.013). After a Bonferroni correction (0.05/3, adjusted alpha = 0.0167) all three of these paired t-tests remained significant, indicating that participants deviated more from the prompt pulse in slower tempo conditions. This suggests that the externally paced pulse is more difficult to maintain and entrain with at slower tempos.

### Deviations in entrainment did not converge on individual SMT

The relationship between the experimental tempo participants deviated towards and SMT was examined by comparing the tempo that participants converged on at the end of the synchronization-continuation task to individual SMT. This was done by averaging participant normalized IOI values from the last 2 seconds of trials, and then taking the difference of a participants SMT and this average. Based on this calculation, values of 0 would reflect a convergence of behavioral entrainment on SMT. Participants did not accurately converge on their SMT as the difference between end of trial tempo and SMT ranged from 10–880 ms (IOI), with a mean difference of 295 ms. The end of trial/SMT difference values were split into high and low rhythmicity groups in each tempo condition, and a mixed model ANOVA was run, with factors group (between-participant, high rhythmicity (n = 9), low rhythmicity (n = 9)), and tempo (within-participant, 50 bpm, 70 bpm, 128 bpm) revealing a main effect of tempo (F(1,2) = 12.93, p < 0.0019). Paired sample t-tests revealed significant differences between 50 and 70 bpm tempo condition and 50 and 128 bpm tempo condition (50–70 bpm, t(17) = 13.13, p < 0.001; 50–128 bpm, t(17) = 3.07, p < 0.007). After a Bonferroni correction (0.05/3, adjusted alpha = 0.0167) these paired t-tests remained significant. The mean of the end of trial/SMT difference was greater in the 50 bpm condition than either the 70 or 128 bpm condition (50 bpm, mean = 461 ms; 70 bpm, mean = 189 ms; 128 bpm, mean = 235 ms), indicating that participants tend to deviate towards SMT from the external pulse in our faster tempo conditions. This is likely due to the proximity of the 70 and 128 bpm tempo conditions to the general range of SMT produced by participants (62–122 bpm).

### Rate of entrainment decay increases with earlier onset of decay, and onset of decay is earlier for individuals with high intrinsic rhythmicity

**Figure 3:**
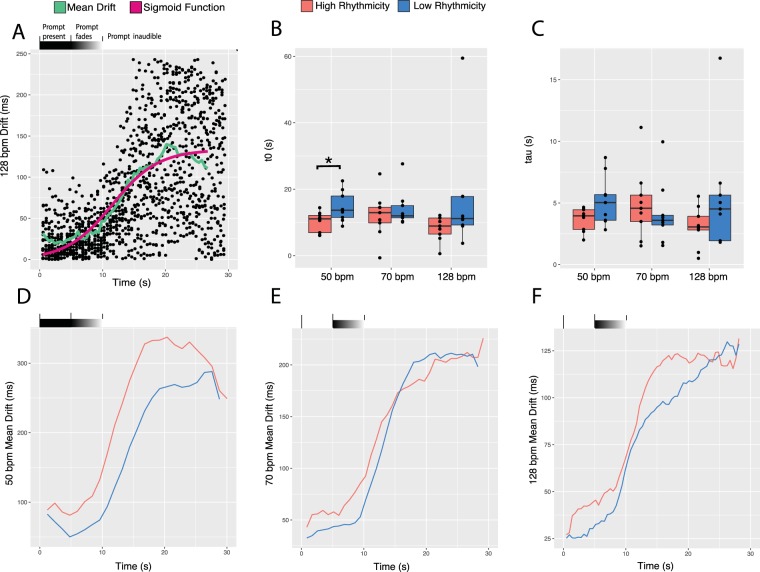
Drift Value analysis. (**A**) The 128 bpm (469 ms IOI) drift value data for one participant plotted along with the condensed mean drift time course and sigmoidal function fitting of that time course. (**B**) t0 coefficients were extracted from the sigmoidal fits and represented the midpoint of the function. These were compared across high and low rhythmicity groups and tempo conditions (50, 70, and 128 bpm; 1200, 857, and 469 ms IOI), revealing a main effect for group that was significant in the 50 bpm (1200 ms IOI) condition. (**C**) Tau coefficients were extracted from the sigmoidal fits and represented the time-constant (inverse of the maximum slope) of the function. No significant findings were found for tau. (**D**–**F**) Plotted time courses for the averaged mean drift time courses for all individuals in the high and low rhythmicity groups at each experimental tempo (50, 70, and 128 bpm; 1200, 857, and 469 ms IOI). See Supplementary Fig. 2 for all participant mean drift time courses plotted together and all participant sigmoidal function fits.

As participants moved from the synchronization to the continuation portion of the synchronization-continuation task, their drum hits became more disorganized relative to the prompt pulse. We here refer to this as entrainment decay, as the stable temporal relationship—i.e. entrainment—between drum hits and prompt beats broke down over time. Drift values were used to assay entrainment decay, as they measure the organization of drum hits relative to the prompt stimulus over the course of a trial. To calculate drift values, participant drum hits were sorted into time windows defined by the midpoint between prompt beats. The absolute value of the difference between drum hit and prompt beat was calculated, making drift values a measure of deviation from the prompt. All drift values for a given participant were averaged together across trials, to produce a time course of mean drift values for each participant (one example drift time course plot shown in Fig. 3A). This time course represents the decay of entrainment from being organized with respect to the prompt—low drift value—to disorganized with respect to the prompt—high drift value. Due to the sorting of participant drum hits into time windows centered on prompt beats, drift values become saturated when drum hits that were initially supposed to align with one prompt beat become closer to another prompt beat—i.e. cross the midpoint between prompt beats—causing them to be sorted into the previous or following time window. This represents temporal disorganization of drum hits relative to the prompt, as now drum hits have a closer temporal relationship with prompt beats they initially were not supposed to align with.

Participants were split into high and low rhythmicity groups and mean drift time courses were averaged together across participants within these groups to produce combined time courses. Three combined time course plots were generated, one for each or the paced tempo conditions, in which the combined time course for high and low rhythmicity individuals were depicted (Fig. 3D–F). This revealed a difference in how entrainment decayed for the high and low rhythmicity groups, moving from organized drumming to disorganized drumming, relative to the prompt. Plots presenting all of the individual participant mean drift time courses for high and low rhythmicity individuals were generated (Supplementary Fig. 2A–C).

Individual mean drift time courses for each participant were fit with a sigmoid function (equation 1; Fig. 3A; Supplementary Fig. 2D–F), from which coefficients t0—representing the midpoint of the fit—and tau—representing the time-constant (inverse of the maximum slope) of the fit—values were extracted. Significant correlations were found between tau and t0 in all three conditions (50 bpm, rho = 0.54, p = 0.023; 70 bpm, rho = 0.55, p = 0.023; 128 bpm, rho = 0.52, p = 0.033), demonstrating that as entrainment decay begins earlier, it occurs at a greater rate. Mixed model ANOVAs were run for both tau and t0 with factors group (between-participant, high rhythmicity (n = 9), low rhythmicity (n = 9)), and tempo (within-participant, 50 bpm, 70 bpm, 128 bpm), producing a main effect for group (F(1,2) = 5.24, p < 0.036) for t0; no main effect was found for group (F(1,2) = 2.29, p = 0.15) for tau. Three two sample t-tests were run comparing high and low rhythmicity groups in each tempo condition, confirming a significant difference in the 50 bpm condition (t(14.025) = −2.48, p = 0.026). After a Bonferroni correction (0.05/3, adjusted alpha = 0.0167) this result was not significant. The lack of main effect for group in tau indicates that the rate of decay dependency on onset of decay is not group specific, while the main effect for group in t0 demonstrates that high rhythmicity individuals began entrainment decay earlier than low rhythmicity individuals. The behavioral differences seen between high and low rhythmicity participants are seen most clearly in the 50 bpm condition (Fig. 3D).

### Distance from SMT does not impact rate and onset of entrainment decay

To assess if the difference in tempo between SMT and the prompt pulse impacted entrainment decay parameters, we subtracted each participants SMT from the tempo of each paced condition (50, 70, and 128 bpm) and took the absolute value. This produced a value that represented the difference in tempo between the participants SMT and each tempo condition. These values were correlated with t0 and tau coefficients separately, producing no significant correlations (t0, 50 bpm: rho = −0.16, p = 0.51/70 bpm: rho = −0.015, p = 0.95/128 bpm, rho = 0.036, p = 0.88; tau, 50 bpm: rho = −0.18, p = 0.47/70 bpm: rho = 0.046, p = 0.85/128 bpm, rho = 0.019, p = 0.94). To asses if intrinsic rhythmicity influences this null finding, tau and t0 correlations were repeated for only participants in high and low rhythmicity groups respectively, again producing no significant correlations (t0, high rhythmicity 50 bpm: rho = −0.28, p = 0.46/low rhythmicity 50 bpm: rho = −0.26, p = 0.49/high rhythmicity 70 bpm: rho = −0.51, p = 0.16/low rhythmicity 70 bpm: rho = 0.22, p = 0.58/high rhythmicity 128 bpm, rho = 0.16, p = 0.68/low rhythmicity 128 bpm, rho = −0.22, p = 0.58; tau, high rhythmicity 50 bpm: rho = 0.23, p = 0.55/low rhythmicity 50 bpm: rho = −0.4, p = 0.29/high rhythmicity 70 bpm: rho = −0.083, p = 0.84/low rhythmicity 70 bpm: rho = 0.05, p = 0.91/high rhythmicity 128 bpm, rho = 0.48, p = 0.19/low rhythmicity 128 bpm, rho = −0.3, p = 0.44). These results indicate that the tempo difference between an eternally paced pulse and SMT to not impact the onset or rate of entrainment decay.

## Discussion

In this study, we sought to characterize synchronization-continuation behavioral entrainment through the examination of multiple experimental tempos (50, 70, and 128 bpm; 1200, 857, and 469 ms IOI) and differences seen in the intrinsic rhythmicity of participants. Intrinsic rhythmicity was assessed through an SMT task where participants drummed at a tempo of their choice. The relative accuracy of the drumming of each participant to their average SMT allowed us to define two groups, high and low rhythmicity individuals. Behavioral differences in synchronization-continuation task entrainment between high and low rhythmicity individuals and tempo conditions were then characterized.

In line with previous synchronization-continuation findings^21,37^, we saw that variability in IOI increases following the transition from synchronization to continuation, and that this effect increased with the tempo of the external prompts. Participants also deviated farther from the external prompt pulse at slower tempos during the continuation portion of our trials, indicating that continuation entrainment is more difficult to maintain at slower tempos, again consistent with the synchronization-continuation literature^21,38,39^. The relationships between high frequencies and increased variability (SD of normalized IOI values), and low frequencies and greater deviation (slope of linear regression fit to normalized IOI values) suggest that continuation processes are tempo dependent and perhaps occur optimally in a range between our two extreme tempo conditions—50 and 128 bpm. SMS studies have defined a behaviorally salient range of IOI for SMS entrainment—200–1800 milliseconds or 33–300 bpm—with musical tempos falling within the boundaries of this range^21^. Greater variability in entrainment accuracy is seen in synchronization-continuation tasks in comparison to SMS tasks^37^ indicating that auditory feedback facilitates error correction and that the mechanisms that underlie continuation entrainment are inherently variable.

In this study, we implemented phase locking values (PLV) to assay participant performance in experimental tasks. PLV are primarily used to compare the synchronization of two different sources in neuroimaging time series and neural spiking data^40^. The SMS literature has previously implemented similar measures to examine the temporal consistency of participant tapping data^41,42^. In this study, PLV acted as a relative metric for how stable entrainment processes are at a constant tempo, allowing us to compare behavioral entrainment performance across different stimulus tempos. We assessed the effects of frequency on entrainment performance, as well as across individuals to examine the effects of intrinsic rhythmicity on entrainment performance.

Our assessment of intrinsic rhythmicity suggests that SMT performance accuracy can predict behavioral entrainment in synchronization-continuation tasks. Participants who showed less variability (as evidenced in higher PLV) in the SMT task were classified as high rhythmicity individuals, whereas participants who showed greater variability (as evidenced in lower PLV) in the SMT task were labeled low rhythmicity individuals. High rhythmicity individuals performed worse in the synchronization-continuation behavioral entrainment tasks compared to low rhythmicity individuals, who exhibited higher accuracy in these tasks. This distinction was obscured in the 70 bpm condition providing further evidence that there is a behaviorally salient tempo range that humans can best coordinate within, as there was a less clear performance bias for SMT consistency vs. externally-paced entrainment tasks at this tempo. These results suggest a difference in entrainment processes, with individuals tending to perform better when either entraining to environmental rhythms or generating endogenous rhythms.

Participants have been shown to exhibit greater entrainment accuracy in synchronization and synchronization-continuation paced at individual participant SMT tasks relative to non-preferred tempos^27,28^ demonstrating that SMT consistency may serve as an indicator of intrinsic rhythmicity and potentially neural processing. Both synchronization-continuation and SMS tasks have been shown to engage different brain areas, suggesting that endogenous neural mechanisms are activated to compensate for a lack of auditory feedback which typically supports entrainment through error correction and predictive processes^43–45^. When participants spontaneously generate pulses in SMT tasks, they would be relying solely on these endogenous mechanisms to produce a pulse, rather than assimilating an external rhythm through the sensory systems. Individual differences in neural processes could then manifest behaviorally in SMT consistency, which would be comparable across participants as behavioral measures would be representative of the endogenous characteristics of an individual. Future research is needed to probe neural correlates of differential performance in SMT and synchronization-continuation tasks, which would allow for the documentation of neural characteristics that underlie differences between high and low rhythmicity individuals.

We found that as participants deviated in tempo over the course of the synchronization-continuation task, they did not converge on their SMT; this is seen most clearly in the 50 bpm tempo condition. The proportionally greater amount of convergence seen in our faster tempo conditions is likely due to the similarity in the 70 and 128 bpm tempo conditions to the general range of SMT produced by participants (62–122 bpm). The limited trial time window during which convergence could occur, in addition to the perceivably larger gap between the 50 bpm tempo and general SMT likely decreased participant convergence on SMT in the 50 bpm condition. Though participants did not accurately converge on SMT tempo, we acknowledge the limitations of our study design in testing this hypothesis and believe that future research might reveal a trend towards convergence.

Differential behavior in the transition from synchronization to continuation entrainment in the synchronization-continuation task was observed based on whether participants were high or low rhythmicity individuals. As the drum hits of a participant transitioned from an organized to a disorganized relationship to the prompt beat—i.e. entrainment decay—high rhythmicity individuals began to drift away from the prompt pulse sooner than low rhythmicity individuals, indicating that high rhythmicity individuals experience an earlier onset of entrainment decay. Though this appears be indicative of the performance bias of high rhythmicity individuals for self-selected pulses over externally dictated pulses, we did not reproduce the previously seen relationship between SMT and entrainment performance in a corresponding synchronization-continuation tempo^27,28^. This could have been due to differential methods of calculating SMT. Delevoye-Turrell *et al*.^28^ used a mobile application to assay SMT outside of the experimental setting while Styns *et al*.^27^ defined SMT using walking pace. SMT has the potential to vary greatly within individuals, and has the potential to be affected by a variety of variables such as mood, arousal, and comfortability. A study by McAuley *et al*.^36^ found that characteristic shifts in SMT do occur over the course of human lifespan and development, but found that consistency in the production of SMT did not vary with age. The mean SMT of the age group that corresponded to the individuals in our study (ages 18–38) was 630 ms (IOI)—roughly 95 bpm—with a standard error of 22 ms—roughly 92–99 bpm. This appears to be a relatively low error in SMT, though in this analysis they excluded all participants who produced standard deviations greater than 25% in SMT and three other un-paced tapping measures, leading to uncertainty of the effects of outliers on these statistics. Our null finding could also be explained by our use of only three external tempos—50, 70, and 128 bpm—in the synchronization-continuation task, whereas Delevoye-Turrell *et al*.^28^ targeted a tempo range around individual SMT and Styns *et al*.^27^ assessed a wide range of tempos with small incremental differences. Temporal consistency of SMT may prove to be a more stable metric of internal rhythmic characteristics.

We also found a correlation between earlier onset of entrainment decay and increased rate of decay across all participants, but did not find a significant difference in rate of decay between high and low rhythmicity individuals. Though decay rate and onset appear to be related, our results indicate that differences in intrinsic rhythmicity do not explain decay rate. We suggest that decay onset could be explained by maintenance of internal auditory imagery representations of rhythm, that decay rate is relatively consistent between high and low rhythmicity individuals, and that decay rate does not depend on these imagery mechanisms. Strong rhythmic imagery would allow low rhythmicity individuals to maintain accurate entrainment for a longer duration, leading to the delayed onset of entrainment decay we characterized for them. Low rhythmicity individuals would preferentially entrain with environmental rhythms, allowing them to develop these enhanced rhythmic imagery abilities.

Neural oscillations have been shown to entrain to periodic auditory stimuli and are hypothesized to support a variety of processes including computational, perceptual, and attentional abilities^46–50^. Multiple neuro-imaging studies have implicated motor system areas—pre-SMA, SMA, cerebellum, PMC, and the basil ganglia—in perceiving a pulse in rhythmic auditory stimuli in the absence of motion^50–58^. These findings, in conjunction with the prevalence of beta rhythms seen in auditory-neural entrainment studies^57,59–61^—as the beta frequency band is implicated in motor preparation and excitation^62,63^—arouses further interest in the unique relationship seen in auditory-motor-neural entrainment.

Neural Resonance Theory (NRT) postulates that neural oscillations entrained to acoustic rhythms represent musical pulse and meter in the brain^64^. A variety of magnetoencephalography (MEG) and electroencephalography (EEG) studies have tested and supported NRT: neural oscillations exhibit anticipatory and sensory responces to periodic auditory stimuli^59,65^; neural oscillations are modulated by imagined accents and meter structures^66,67^; the phase of neural oscillations influences behavioral responses to isochronous stimuli^68^; auditory stimuli with no net energy at pulse frequency elicit responses in frequency bands that correspond with pulse and meter^69,70^. These findings demonstrate that the pulse and rhythm can be endogenously generated and represented in neural oscillatory activity. Collectively there is strong evidence that periodic auditory rhythms can drive neural and motor activity, and that rhythmic entrainment may be responsible for many of the health, developmental, and cognitive benefits conferred by music, and in turn music therapy interventions^1,10,71,72^.

Studies have linked increases in amplitude of entrained oscillations^42,73^ and alpha band activity^73,74^ to performance increases and decreases in behavioral entrainment respectively. Henry *et al*.^73^ observed that entrained oscillations and alpha activity impacted behavioral performance oppositely within the same population performing a paced gap detection task, suggesting that these processes differentially modulate entrainment and influence selective attention to external rhythms. These mechanisms could underlie our findings, with high rhythmicity individuals more successfully exploiting internal alpha band associated endogenous processes to generate and maintain SMT. Low rhythmicity individuals may have exhibited stronger entrained oscillatory activity during synchronization, allowing them to maintain rhythmic imagery longer during the transition to continuation and produce the characteristic delayed onset of entrainment decay we associated with them. Lakatos *et al*.^75^ demonstrated an alternation between increased alpha oscillation power and entrained oscillatory activity to external rhythms in a continuous auditory selective attention task in non-human primates. Task behavioral performance increased during the entrained oscillation periods and decreased during the heightened alpha power periods. This further supports the differential implication of entrained oscillatory activity and alpha oscillations in entrainment to external rhythms and endogenous processes.

Other work has demonstrated that entrainment response of endogenous oscillations to brain stimulation is state-dependent, with greater endogenous oscillatory power causing a reduced influence of stimulation^76,77^. We speculate a similar phenomenon here could underlie our high and low rhythmicity entrainment performance differences. High intrinsic rhythmicity individuals could have greater endogenous oscillatory power, facilitating temporally stable SMT production in the absence of an external pulse, while low intrinsic rhythmicity individuals could have reduced oscillatory power, leading to greater entrainment of external pulses and improved performance in synchronization-continuation tasks. As the neural correlates of high and low rhythmicity groups have not been investigated, future work is needed to explore this hypothesis.

Our results pose implications for music therapy professionals and intervention implementations. Evidence of a behaviorally optimized range of tempos that individuals best entrain with suggests that the standardization and consideration of tempo variability in music therapy interventions could benefit clinical practice^8,78^. Results demonstrating the salience of intrinsic rhythmicity show that rhythmic processes can be dictated by endogenous differences. Clinicians could use SMT tasks to assess the entrainment ability and affinity of a patient for certain tempos, allowing rhythmic interventions to be individually designed to target specific deficits. SMT tasks vs. externally paced entrainment tasks could be used to engage differential processes that underlie entrainment. The variability in behavioral entrainment responses to different tempo conditions indicate that intentionally pacing rhythmic interventions at optimal tempos could confer therapeutically consistency. In contrast, designing an intervention at a physiologically taxing rapid tempo could target physiological coordination abilities, whereas slower tempos could challenge the predictive and anticipatory mechanisms that would target neurology. Future research would be needed to assess the effects of differentially targeted tempo interventions. These hypothetical parameters for rhythmic intervention design propose novel pathways through which music therapy interventions can operate. Our results are suggestive of the ability of rhythm to target a range of mind-body sensorimotor processes that can be translated into standardized music therapy interventions for clinical use. After development, these interventions would need to undergo rigorous trials to better understand the specific mechanisms that underlie music therapy interventions and to support the evidence-based practice of music therapy.

In sum, we used behavioral entrainment performance in an SMT task to characterize high and low intrinsic rhythmicity individuals. Group assignment predicted behavioral entrainment performance in synchronization-continuation tasks at various tempos. High rhythmicity individuals were more successful at accurately generating and maintaining consistent SMT while low rhythmicity individuals were more successful at entraining with externally paced pulses. These distinctions were seen most clearly and consistently in the slowest tempo condition, indicating that the pulse frequency impacts behavioral entrainment and the salience of performance differences between high and low rhythmicity individuals. Our findings are suggestive of two underlying neural mechanisms that differentially engage entrainment processes. These findings have relevance for the field of music therapy and the development of targeted interventions for therapeutic treatment.

## Methods

### Participants

Twenty adults (5 males and 15 females, 19 right handed, 1 left handed) participated in this experiment for a $15 compensation fee. Participants ranged in age from 18 to 26, had command of the English language, and had no history of cardiovascular or neurological diseases. None of the participants were taking medications for central nervous system disorders. Participants were non-musically trained, having at most three years of music training before the age of 12 years old. Those with continued training beyond the age of 12 were excluded from participating. These measures were taken to control for and limit musical training, which has been shown to affect rhythmic ability^71^. All participants provided written informed consent before participating and were recruited from the Chapel Hill area. The study was conducted within an isolated studio room in Keenan Music Building at the University of North Carolina at Chapel Hill (UNC). The UNC Institutional Review Board approved this study. All methods were performed in accordance with the relevant guidelines and regulations.

### Experimental tasks

**Figure 4:**
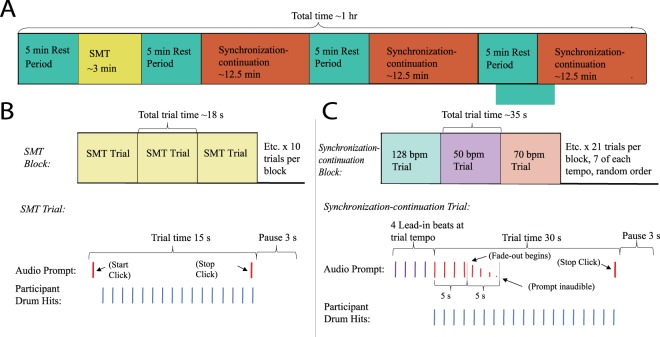
Study and task design. (**A**) Each participant participated in an SMT task block and three synchronization-continuation task blocks that were all preceded by and interspersed with 5-minute resting periods. (**B**) The SMT task block consisted of 10 identical SMT task trials that were presented in succession. (**C**) The synchronization-continuation task block consisted of 21 trials—7 at each of the three experimental tempos (50, 70, and 128 bpm; 1200, 857, and 469 ms IOI)—presented in a random order.

This study contained two experimental tasks. For clarity, we here define “drum hits” as the behavioral responses participants produce in these tasks, “prompt beats” as the auditory stimuli presented in the synchronization-continuation task, “tempo” as the frequency or speed at which drum hits are played or prompt beats are presented (defined in bpm or IOI ms), and “pulse” as the isochronous rhythm (either drum hits or prompt beats) that participants entrain with or produce. Participants were seated 4 feet in front of a pair of stereo speakers that were adjusted to chair height on a stand. The speakers presented the prompt stimuli and cues throughout the experimental tasks at a volume of 60 decibels SPL. The first task examined the individual intrinsic rhythmicity of each participant through a spontaneous motor tempo (SMT) task (Fig. 4B). Participants were instructed to hit the drum sustaining a constant pulse at their own, naturally comfortable tempo. Each SMT task trial lasted 15-seconds in duration and was repeated for a total of 10 trials. A 3-second pause occurred between consecutive trials. Start-stop cues to begin and end each trial consisted of a high-pitched “start” click followed by a lower-pitched “stop” click, emitted from external speakers.

The second task investigated the entrainment of participants to external prompt pulses at three experimental tempos—50, 70, and 128 bpm; or 1200, 857, and 469 milliseconds (IOI)—through a synchronization-continuation task (Fig. 4C). These experimental tempos were chosen to cover a range of common musical pulses that were not physiologically challenging to produce and could be utilized in a rhythmic music therapy context^8,78^. The two slower tempos—50 and 70 bpm—are commonly used in music therapy as they are easy for individuals to synchronize with and are close in frequency to other physiological rhythms such as heart rate^8,78^. The 128 bpm tempo was used as a faster control tempo, allowing us to see if tempos that are faster that those commonly used in music therapy impact entrainment processes. The 128 bpm tempo also functioned as a non-harmonic control tempo, as interactions between stimulus frequencies and their harmonics are documented in the entrainment literature^42,70^.

In the synchronization-continuation task, presented prompt beats defined a pulse which participants were directed to drum with continuously in real time, copying and maintaining the prompt pulse at the same tempo on the drum. Four lead-in start clicks prefaced the task and alerted participants to the tempo condition they were to entrain with. Lead-in clicks seamlessly transitioned into the prompt beats at the same tempo, and participants were asked to begin drumming in time with the first prompt beat. This allowed participants to orient themselves to the experimental tempo before the task began and data were generated. The lead-in clicks were presented at a higher pitch than the prompt beats to help distinguish the two and cue the participant to start drumming. Trials were 30 seconds long, and began with the first prompt beat. Five seconds into the 30 second trial, the prompt began to decrease evenly in volume, becoming inaudible over the course of 5-seconds. The participant continued drumming non-stop, at the same pulse in which this trial began, until the low pitched “stop” click was presented. A 3-second pause occurred between each trial. Each tempo condition was presented 7 times in a random manner within a block, resulting in 21 total trials per block.

### Experimental measures

Two data streams—the presented prompt beats and drum hits—were digitized and recorded continuously over the course of the experimental session. Participants played a 10” hand-held drum by holding the drum vertically against their upper leg with the hand of their preference and hitting it with a mallet held in the other hand. Drum hits were recorded through a piezoelectric transducer attached to the membrane of the drum. Data were recorded continuously from these sources with a DAQ (NI USB DAQ 6001) for the duration of the experiment at a sampling rate of 1000 Hz. A custom MATLAB (Mathworks, Inc. R2016a) code was written to manage data acquisition.

Questionnaires assessing attention, handedness, task load, and musical background were administered. Qualitative measures were also taken in the form of observational notes recorded by the experimenter over the duration of the experimental session.

### Procedure

Upon arrival, participants were seated in a chair facing a stereo speaker set. After signing a consent form and completing the attention and handedness questionnaires, data collection was started and ran continuously for the duration of all experimental tasks.

The SMT task was administered, followed by three identical blocks of the synchronization-continuation task. Five-minute resting periods occurred before the start of each task. During this, the participants were given reading material unrelated to the study that was then collected at the end of the resting period. Resting periods were designed to give participants a break from the monotonous drumming tasks.

Upon completion of the third synchronization-continuation task block, data collection was ceased. After completing the task-load and musical background questionnaires, participants collected their compensation and were allowed to leave. Total participation time for each individual averaged 90 minutes.

### Data analysis

Data were analyzed using custom MATLAB and R scripts. SMT task data were excluded in 2 participants due to data acquisition errors. The data for each participant were visually screened for errors or noise. Trials where data either were not recorded or where the signal to noise ratio was poor were discarded. A total of five trials were discarded across all participants. The remaining data for each participant were broken down into trials and sorted into four groups, one for the SMT task and one for each of the three experimental tempo conditions in the synchronization-continuation task. Trial beginnings were time locked with the start click and continued for 15 seconds in the SMT task, and were time locked with the first prompt beat and in the synchronization-continuation task, continuing for 30 seconds.

A peak localization function was written to identify the onset times of drum hits and prompt beats, and a quality control analysis was conducted on the prompt beat and drum hit trial data for each participant to ensure that peak time points could be labeled with a maximum error of 6-miliseconds.

Raster plots were generated from the drum hit data for all trials for a participant to visually screen the data. The peak height parameter in the peak localization function was optimized for each participant to increase the number of detected drum hits without mislabeling non-drum hit noise as a hit.

SMT was calculated per participant by finding the number of drum hits per trial, dividing this by the time elapsed between the first and last drum hits in a trial, and averaging these trial values together to produce a beats-per-minute value representing average pace that participants spontaneously produced.

Phase locking values (PLV) were calculated for each drum hit relative to the nearest previous prompt beat. Each hit was represented as a vector in the polar plane. Phases angles 0 to 2 pi were assigned to each drum hit depending on where it occurred relatively between the previous prompt beat and the following one; each vector was assigned a magnitude of 1. Vectors were averaged together to yield PLV. Temporally organized responses produce a larger PLV as the majority of vectors occur at the same phase angle and average together to produce a larger value. A lower PLV reflects temporally disorganized data, as greater variability in phase angle lowers the directional magnitude during the averaging of response vectors. The prompt signal was extrapolated for the duration of all synchronization-continuation task trials so that drum hits could be paired with the prompt beat. For the SMT task trials the average SMT of each participant was used as the reference signal and extrapolated across each trial. Time extrapolation plots were generated for trials per participant to ensure that the maximum error in the extrapolation was 7-miliseconds. PLV was only calculated from drum hits in the second half of each trial. This was done to prevent the paced stimulus from altering our results in the synchronization-continuation task as the prompt had faded out by this point, and to give participants adequate time to entrain with either their maintained pulse in the synchronization-continuation task or settle into a stable pulse in the SMT task.

Inter onset intervals (IOI) were calculated from the duration between each drum hit and the nearest previous detected hit. Dropped hits described earlier resulting from the peak detection function produced IOI values that were not representative of participant performance and thus were excluded from the analysis. IOI values greater than 1600, 1300, and 700 ms were excluded from the 50, 70 and 128 bpm conditions respectively. The exclusion criterion was predicated on a visual screening of the raw data, raster plots, and IOI values. All excluded points resulted from dropped hits from the peak localization function. IOI values were normalized with respect to tempo by subtracting the time in milliseconds of the prompt IOI from each IOI value and dividing by the prompt IOI.

Drift values were calculated to measure the organization of drum hits relative to the prompt stimulus over the course of a trial. The prompt stimulus was extrapolated for the duration of trials, and drum hits were sorted into time windows defined by the midpoint between prompt beats. The difference between the time point of a drum hit and the time point of a prompt beat was taken to calculate the drift value, resulting in one value for each drum hit. The absolute value of each drift value was taken, so that hits that occurred before the prompt beat would be comparable to hits that came after the prompt beat, making drift exclusively a measure of drum hit deviation from the prompt. Placeholder values were used to align the initial drift value in a trial relative to the initial drift values in other trials, as there was variability in which prompt beat the first drum hit a participant played in a trial aligned with. These were non-numerical values that were not included in the drift value calculations. Placeholders were also used to account for hits dropped by the peak localization function, as previously discussed. The same exclusion criteria were used to identify dropped hits and fill them in with placeholders. Drift values for each participant were averaged together for each participant to produce mean drift time courses. These mean drift time courses were fit with sigmoid functions (equation 1):

1 $$y=\frac{a}{(1+{e}^{\frac{-(x-t0)}{tau}})}$$

In this equation, the tau parameter represents the time constant (inverse of the maximum slope), t0 represents the midpoint, and $a$ represents the amplitude of the fitted equation.

Statistical analyses were performed and figures were generated in R using custom scripts and “ggplot2”, “ggpubr”, and the “ez” packages. Mixed model ANOVAs with a three group within participants factor and a two group between participants factor were used in most cases to determine significance. Mauchly’s Test for Sphericity was used to be sure repeated measures did not violate the sphericity assumption, and Greenhouse-Geisser correction values were used to adjust p-values. Other statistical tests used included one-way repeated measures ANOVAs with a three group within participants factor, linear regressions, spearman correlations, and paired and two sample t-tests. Bonferroni corrections were used to control ANOVA post-hoc testing. Significance designations in figures indicate results obtained from uncorrected tests. The datasets generated during and/or analyzed during the current study are available from the corresponding author on request.

## Electronic supplementary material

Supplementary Information
