# Supplementary material for: Intrinsic Rhythmicity Predicts Synchronization-Continuation Entrainment Performance
Source: Sci Rep. 2018 Aug 6;8:11782. doi: 10.1038/s41598-018-29267-z (PMC6079093; doi:10.1038/s41598-018-29267-z)
Supplement: Supplementary file 1 — Supplementary Information [file 41598_2018_29267_MOESM1_ESM.pdf]

Intrinsic Rhythmicity Predicts Synchronization-Continuation Entrainment Performance

Trevor McPherson[1,6], Dorita Berger[1], Sankaraleengam Alagapan[1,6], and Flavio Fröhlich[1,2,3,4,5,6,7]

Correspondence should be addressed to: Flavio Fröhlich, 115 Mason Farm Rd. NRB 4109F, Chapel Hill, NC. 27599. Email: flavio_frohlich@med.unc.edu

1 Department of Psychiatry, University of North Carolina at Chapel Hill, Chapel Hill NC 27599, USA

2 Department of Neurology, University of North Carolina at Chapel Hill, Chapel Hill NC 27599, USA

3 Department of Biomedical Engineering, University of North Carolina at Chapel Hill, Chapel Hill NC 27599, USA

4 Department of Cell Biology and Physiology, University of North Carolina at Chapel Hill, Chapel Hill NC 27599, USA

5 Neuroscience Center, University of North Carolina at Chapel Hill, Chapel Hill NC 27599, USA

6 Carolina Center for Neurostimulation, University of North Carolina at Chapel Hill, Chapel Hill NC 27599, USA

Supplementary Tables and Figures

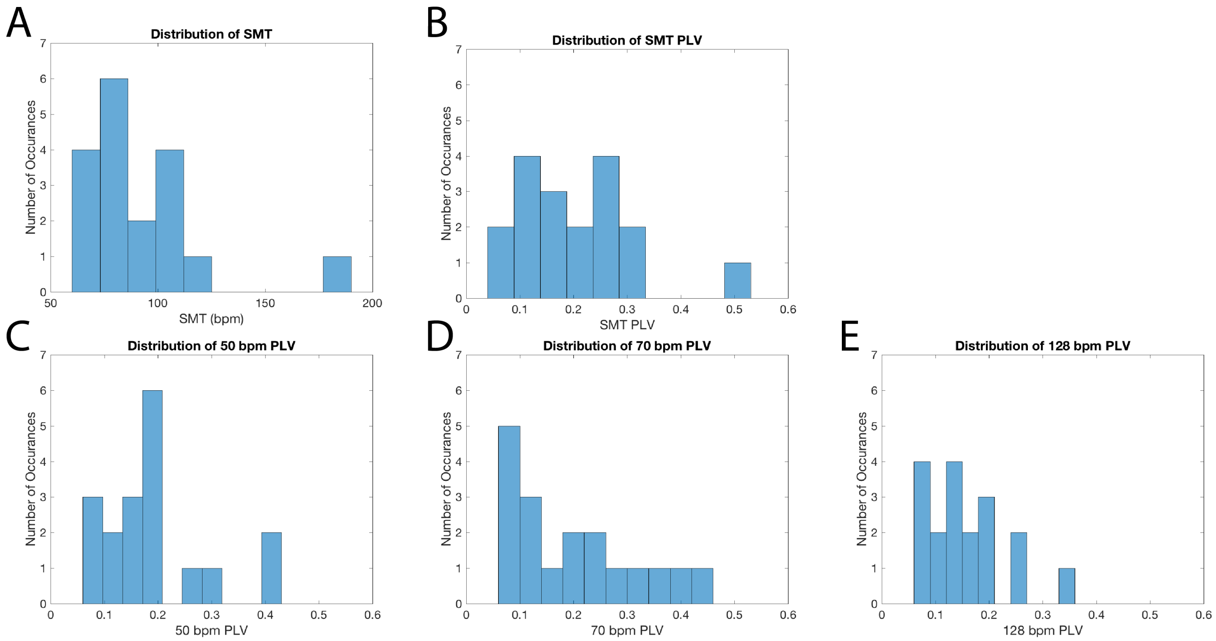

Supplementary Figure 1

A. Distribution plot of participant SMT.

B, C, D, & E. Distribution plots of participant PLV data in the SMT task and the 50, 70, and 128 bpm (1200, 857, and 469 ms IOI) synchronization-continuation task.

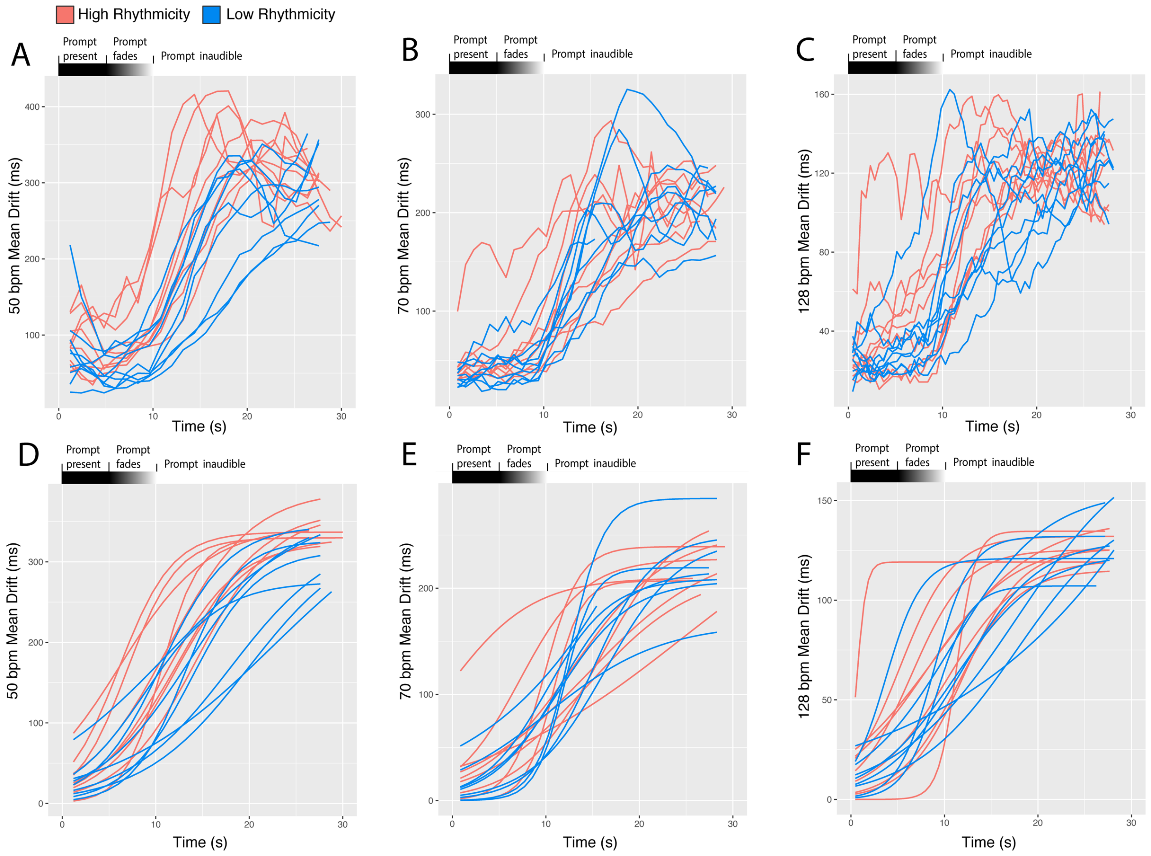

Supplementary Figure 2

A, B, & C. Mean drift time courses plotted for each participant, color coded for high and low rhythmicity individuals for each tempo condition (50, 70, and 128 bpm; 1200, 857, and 469 ms IOI).

D, E, & F. Sigmoidal fits plotted for each participant, color coded for high and low rhythmicity individuals.
